# Effectiveness of Copeptin, MR-proADM and MR-proANP in Predicting Adverse Outcomes, Alone and in Combination with Traditional Severity Scores, a Secondary Analysis in COVID-19 Patients Requiring Intensive Care Admission

**DOI:** 10.3390/jcm13072019

**Published:** 2024-03-30

**Authors:** Emanuele Varaldo, Francesca Rumbolo, Nunzia Prencipe, Fabio Bioletto, Fabio Settanni, Giulio Mengozzi, Silvia Grottoli, Ezio Ghigo, Luca Brazzi, Giorgia Montrucchio, Alessandro Maria Berton

**Affiliations:** 1 Division of Endocrinology, Diabetology and Metabolism, Department of Medical Sciences, University of Turin, 10126 Turin, Italy; 2 Clinical Chemistry and Microbiology Laboratory, S. Croce and Carle Cuneo Hospital, 12100 Cuneo, Italy; 3 Division of Clinical Biochemistry, Department of Laboratory Medicine, University of Turin, 10126 Turin, Italy; 4 Department of Surgical Sciences, University of Turin, 10126 Turin, Italy; 5 Anestesia e Rianimazione 1 U, Department of Anesthesia, Intensive Care and Emergency, Città della Salute e della Scienza Hospital, 10126 Turin, Italy

**Keywords:** CT-proAVP, adrenomedullin, atrial natriuretic peptide, SOFA, MuLBSTA, SAPS II

## Abstract

**Objective:** To investigate whether copeptin, MR-proADM and MR-proANP, alone or integrated with the SOFA, MuLBSTA and SAPS II scores, are capable of early recognition of COVID-19 ICU patients at increased risk of adverse outcomes. **Methods:** For this predefined secondary analysis of a larger cohort previously described, all consecutive COVID-19 adult patients admitted between March and December 2020 to the ICU of a referral, university hospital in Northern Italy were screened, and clinical severity scores were calculated upon admission. A blood sample for copeptin, MR-proADM and MR-proANP was collected within 48 h (T1), on day 3 (T3) and 7 (T7). Outcomes considered were ICU and in-hospital mortality, bacterial superinfection, recourse to renal replacement therapy (RRT) or veno-venous extracorporeal membrane oxygenation, need for invasive mechanical ventilation (IMV) and pronation. **Results:** Sixty-eight patients were enrolled, and in-hospital mortality was 69.1%. ICU mortality was predicted by MR-proANP measured at T1 (HR 1.005, 95% CI 1.001–1.010, *p* = 0.049), although significance was lost if the analysis was adjusted for procalcitonin and steroid treatment (*p* = 0.056). Non-survivors showed higher MR-proADM levels than survivors at all time points, and an increase in the ratio between values at baseline and at T7 > 4.9% resulted in a more than four-fold greater risk of in-hospital mortality (HR 4.417, *p* < 0.001). Finally, when considering patients with any reduction in glomerular filtration, an early copeptin level > 23.4 pmol/L correlated with a more than five-fold higher risk of requiring RRT during hospitalization (HR 5.305, *p* = 0.044). **Conclusion:** Timely evaluation of MR-proADM, MR-proANP and copeptin, as well as changes in the former over time, might predict mortality and other adverse outcomes in ICU patients suffering from severe COVID-19.

## 1. Introduction

The COVID-19 (coronavirus disease 19) pandemic has posed an unparalleled challenge for healthcare systems worldwide. Among these, the Italian healthcare system faced significant strain right from the onset of the crisis. Even though the SARS-CoV-2 virus can sometimes clinically manifest itself in severe forms, its ability to spread at a rapid rate can greatly increase the number of patients that may require hospitalization, quickly filling the capacity of hospital wards and intensive care units (ICUs) [1].

Thus, the management of the different kinds of patients is far from simple: indeed, some may just need medical therapy and oxygen support, others more invasive procedures, and still others may develop even more severe complications that usually require further and more complex interventions [2,3]. Certainly, amidst various clinical presentations, the SARS-CoV-2 virus can lead to an intense cytokine storm, causing various complications including acute respiratory distress syndrome (ARDS) and sometimes even shock [4,5]. As of today, several predictors of disease severity, including markers of inflammation and tissue damage, blood gas analysis and radiological characteristics of lung infiltrates [6,7,8,9], have been proposed. However, the progression of viral infections such as COVID-19 is sometimes extremely rapid [10], making it difficult to allocate and organize the appropriate hospital resources and medical care needed for each of these different situations beforehand.

The attempt to find a possible correlation between some endocrinological markers and COVID-19 severity has already been investigated in the literature with encouraging results [11,12,13,14,15]. For sure, in light of the longer half-life and stability of mid-regional pro-adrenomedullin (MR-proADM), copeptin and mid-regional pro-atrial natriuretic peptide (MR-proANP), with respect to their biologically active counterpart (namely ADM, AVP—arginine vasopressin—and ANP), the research focus has been shifted especially to these three markers. In particular, ADM exerts a marked anti-inflammatory activity by inhibiting the synthesis of certain inflammatory cytokines and, on the other hand, favoring endothelial stability [16]. AVP has a key role in regulating the function of the hypothalamic–pituitary–adrenal (HPA) axis as well as in the transition from innate to adaptive immunity, carried out through the V1b receptors expressed in the thymus [17]. Finally, ANP is involved in a reduction in peripheral resistances (a process in which also ADM and AVP take part [18]) which in turn is partly responsible for the pathogenesis of shock [19]. Furthermore, these biomarkers are associated with morbid conditions such as sepsis or pulmonary embolism (PE) and more generally with endothelial damage, thus characterizing multiorgan failure in severe viral infections and septic shock [20,21,22,23,24].

From a pathophysiological standpoint, it is thus evident that variations in the concentrations of these markers could greatly impact the clinical evolution, sometimes dramatically fast, of patients affected by COVID-19.

Based on these premises, in the present secondary analysis of a larger population previously described [14,25], we aimed at evaluating MR-proADM, copeptin and MR-proANP as promptly available prognostic markers, both alone or in combination with some of the commonly used mortality scores, such as SOFA (Sequential Organ Failure Assessment), SAPS II (Simplified Acute Physiology Score II) and MuLBSTA (Multilobular infiltration, hypo-Lymphocytosis, Bacterial coinfection, Smoking history, hyper-Tension and Age). SOFA and SAPS II are typically used to predict ICU mortality, while MuLBSTA predicts 90-day mortality in viral pneumonia [26,27,28]; all require rather long calculation times and provide an optimizable accuracy [29].

The primary endpoint was to identify endocrine biomarkers capable of predicting ICU and in-hospital mortality as well as recognizing patients at higher risk of complications like bacterial superinfection, or the need for renal replacement therapy (RRT).

## 2. Materials and Methods

This was a predefined secondary analysis from a larger cohort that was previously described [14,25], aimed at examining in detail the prognostic role of copeptin and MR-proANP together with MR-proADM in COVID-19 ICU patients.

All consecutive patients hospitalized for COVID-19 between 1 March 2020 and 31 December 2020 in the university ICU of Città della Salute e della Scienza Hospital of Turin (Italy) were screened. Access to the ICU could be preceded by access to the emergency department or hospitalization in a COVID-19 department of the same hospital or of another ICU in Piedmont (Northern Italy).

The inclusion criteria were as follows: (1) admission in the mentioned ICU; (2) ongoing infection by SARS-CoV-2 confirmed by molecular nasopharyngeal swab or bronchoalveolar lavage fluid; (3) age > 18 years; (4) availability of plasma samples at T1 (first 48 h from ICU admission), T3 (between 49 and 96 h after ICU admission) and T7 (between 6 and 8 days after ICU admission) for measurement of MR-proADM, copeptin and MR-proANP levels; (5) availability of the parameters, collected at the time of admission to the ICU, necessary to calculate the prognostic scores SOFA, SAPS II and MuLBSTA.

On the other hand, no specific exclusion criteria were adopted. The study followed the STROBE statement for reporting observational studies [30].

The study was approved by the Local Ethics Committee (cod. 0069865, 21 July 2020), and it was conducted in accordance with the principles of the Declaration of Helsinki. Written informed consent was obtained in all compatible cases, and waived in other cases, in accordance with the local Ethics Committee’s Italian regulation.

### 2.1. Data Collection

For all patients, information about age, sex, height, weight and body mass index (BMI) was collected. For each patient, the date of admission into the ICU and any dates of discharge from the hospital, transfer to another ward or death as well as data regarding administration of corticosteroid, tocilizumab and hydroxychloroquine were collected; the length of hospitalization for each patient, differentiating the subjects discharged from those who died, was registered.

For each patient, at the time of admission to the ICU, SOFA, SAPS II and MuLBSTA scores were calculated. Blood samples were collected at three different time points:

- T1: first 48 h from the moment of admission to the ICU;
- T3: between 49 and 96 h after the moment of admission to the ICU;
- T7: between 6 and 8 days after the moment of admission to the ICU.

At these times, MR-proADM, copeptin, MR-proANP, c-reactive protein (CRP), procalcitonin (PCT) and eGFR (estimated glomerular filtration rate, calculated with the formula of Chronic Kidney Disease Epidemiology Collaboration, CKD-EPI) were evaluated.

The selected time points were chosen considering that T1 represented the first comprehensive biochemical evaluation conducted upon ICU admission, and likewise, T3 was the subsequent evaluation immediately thereafter. As for T7, this time point was consistent with those previously collected in the main analyses [14,25].

Finally, data about any recourse to pronation, invasive mechanical ventilation (IMV) and veno-venous extracorporeal membrane oxygenation (vv-ECMO) were collected. Superinfection was defined as a bacterial infection occurring more than 48 h after ICU admission [31].

### 2.2. Determination Methods

All biochemical measurements were performed with automated assays in the same laboratory (Laboratory of Clinical Biochemistry, A.O.U. Città della Salute e della Scienza Hospital of Turin).

In particular, the concentrations of MR-proADM, copeptin and MR-proANP were determined with the B.R.A.H.M.S. automated method KRYPTOR compact PLUS^®^ (Thermo Fisher Scientific, Hennigsdorf, Germany), which uses the TRACE (Time-Resolved Amplified Cryptate Emission) technique. The detection limit of the assay was 0.05 nmol/L for MR-proADM, 0.9 pmol/L for copeptin and 0.05 pmol/L for MR-proANP; the intra- and inter-assay coefficients of variation were <4% and <11% for MR-proADM, <7% and <12% for copeptin, and <4% and <11% for MR-proANP, respectively.

To assess changes in biomarkers over time, ratios at the various study times (T3/T1, T7/T1 and T7/T3) were also calculated.

### 2.3. Statistical Analysis

Non-normally distributed variables and categorical data were expressed as median and interquartile range [IQR] and counts and percent, respectively. Comparisons between patient groups at different study times were performed using non-parametric tests such as the Mann–Whitney test and the Kruskal–Wallis rank sum test for independent samples. The Wilcoxon matched-pairs signed-rank test and Friedman test were used to identify differences between paired samples. The chi-square test and Fisher’s exact test were used to evaluate the association between binary variables, while Spearman’s test was used to evaluate the correlation between continuous ones. Univariate and multivariate logistic regression models were calculated to define the association between the different variables and to assess the accuracy of integration between the scores and biomarkers in predicting the outcome of interest. The Kaplan–Meier method was used to compare survival curves between two or more groups. Receiver operating characteristic (ROC) analysis was used to calculate cut-offs with maximum sensitivity (Se) and specificity (Sp) for biochemical parameters. The multivariate Cox regression model was used to evaluate the impact of the variables analyzed and to assess the accuracy of integration between scores and biomarkers in predicting the outcome of interest. Given the sample size was not sufficiently large and to avoid potential overfitting, several multivariate regression models were created, considering demographic features (sex and age), metabolic comorbidities (diabetes mellitus, arterial hypertension and obesity), inflammatory indices (CRP and PCT), immunomodulation treatments (corticosteroids, tocilizumab and hydroxychloroquine) and prognostic scores (SOFA, SAPS II and MuLBSTA).

A cut-off of *p*-value < 0.05 was considered as statistically significant. Statistical analysis was performed using MedCalc^®^ (Statistical Software version 20.007, MedCalc Software Ltd., Ostend, Belgium). Figures were created using GraphPad Prism^®^ (version 8.0.2; GraphPad Software Inc., La Jolla, CA, USA).

## 3. Results

**Figure 1:**
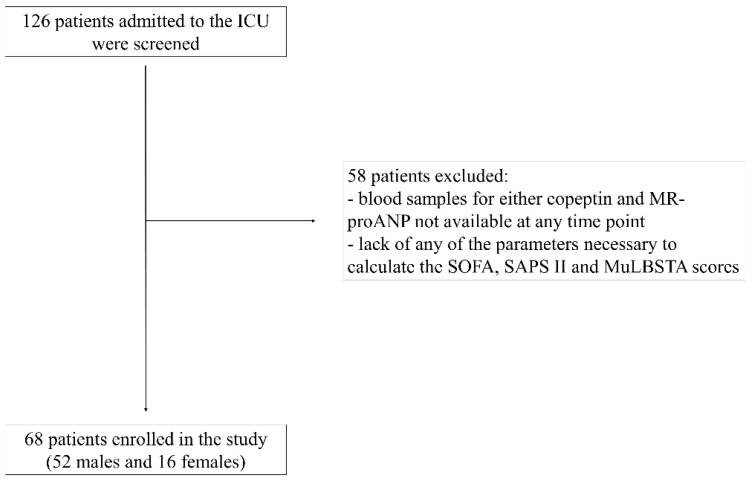
Enrollment process flowchart. ICU: intensive care unit; MR-proANP: mid-regional pro-atrial natriuretic peptide; SOFA: sequential organ failure assessment; SAPS II: simplified acute physiology score II; MuLBSTA: multilobular infiltration, hypo-lymphocytosis, bacterial coinfection, smoking history, hyper-tension and age.

Between 1 March 2020 and 31 December 2020, 126 consecutive patients hospitalized for COVID-19 were screened. Amongst them, 58 patients were later excluded because plasma samples for either copeptin or MR-proANP at the different time points were not available and because of the lack of the parameters necessary to calculate the aforementioned prognostic scores (Figure 1). In the end, 68 patients (52 males and 16 females, median age 63 [56–71] years) with a BMI of 28 [26–32.5] kg/m^2^ who met the inclusion criteria were enrolled in the study.

In our population 43 subjects were obese (63.2%), 12 subjects were affected by diabetes mellitus (17.6%) and 45 subjects were affected by arterial hypertension (66.2%). The median length of stay (LOS) in ICU was 13 [7–22] days, while the median LOS in hospital was 20 [13–30] days. Demographic, clinical and biochemical characteristics of the enrolled patients are listed in Table 1.

### 3.1. Mortality

Forty-seven patients (69.1%) died during hospital stay with a median of days between ICU entry and date of death of 16 [10–26] days. ICU mortality was 61.8% (42/68) since only 5 patients out of 47 died after being discharged from ICU. Amongst the different mortality scores, SOFA was the best in predicting ICU mortality (HR 1.122, 95% CI 1.025–1.228, *p* = 0.012).

ICU mortality was significantly predicted by MR-proANP measured at T1 as well, even if adjusted for demographic features, metabolic comorbidities, CRP at T1 and immunomodulation therapies (HR 1.005, 95% CI 1.001–1.010, *p* = 0.049) (Table 2). Statistical significance was lost if the analysis was adjusted for PCT and concomitant corticosteroid treatment (*p* = 0.056).

**Figure 2:**
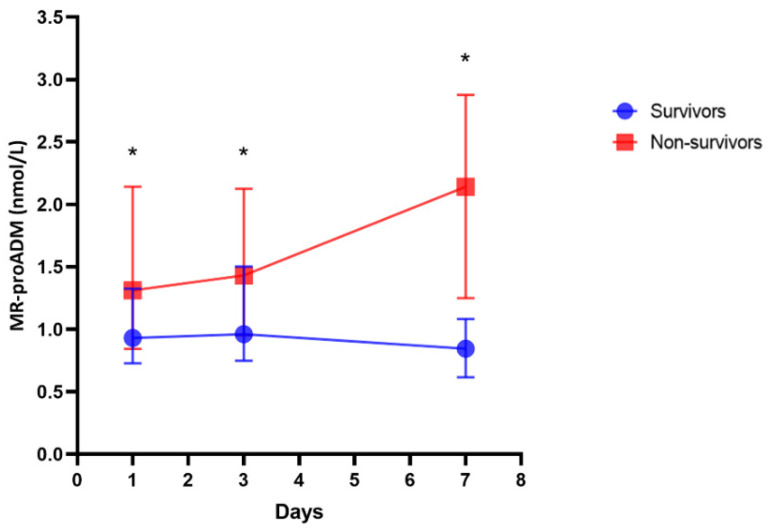
Trend of mid-regional pro-adrenomedullin (MR-proADM) over time in survivors and non-survivors. * *p* < 0.05 at every time point.

MR-proADM measured at every time point was significantly higher in non-survivors compared to survivors (Figure 2, Table 3).

**Figure 3:**
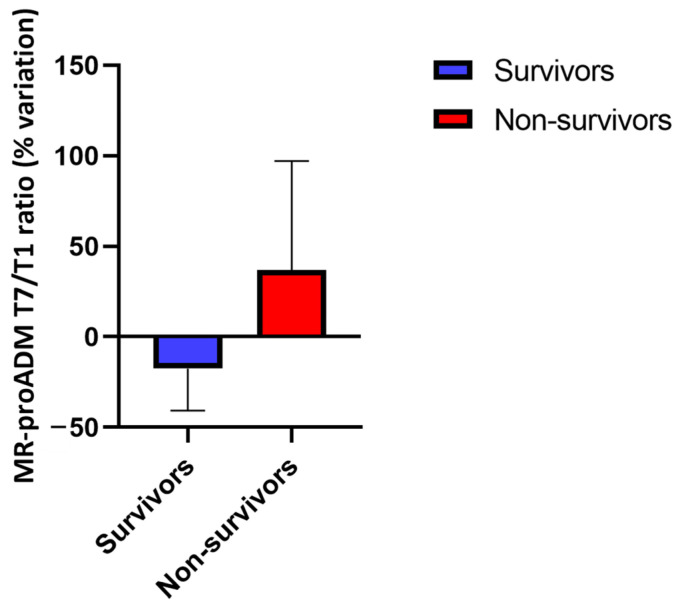
Percentage variation of mid-regional pro-adrenomedullin (MR-proADM) between T1 (first 48 h after the moment of admission to the ICU) and T7 (between 6 and 8 days after the moment of admission to the ICU) in survivors and non-survivors. ICU: intensive care unit.

Accordingly, the change in MR-proADM values between T1 and T7 differed between non-survivors and survivors (+36.9 vs. −17.6%, *p* < 0.001) (Figure 3).

**Figure 4:**
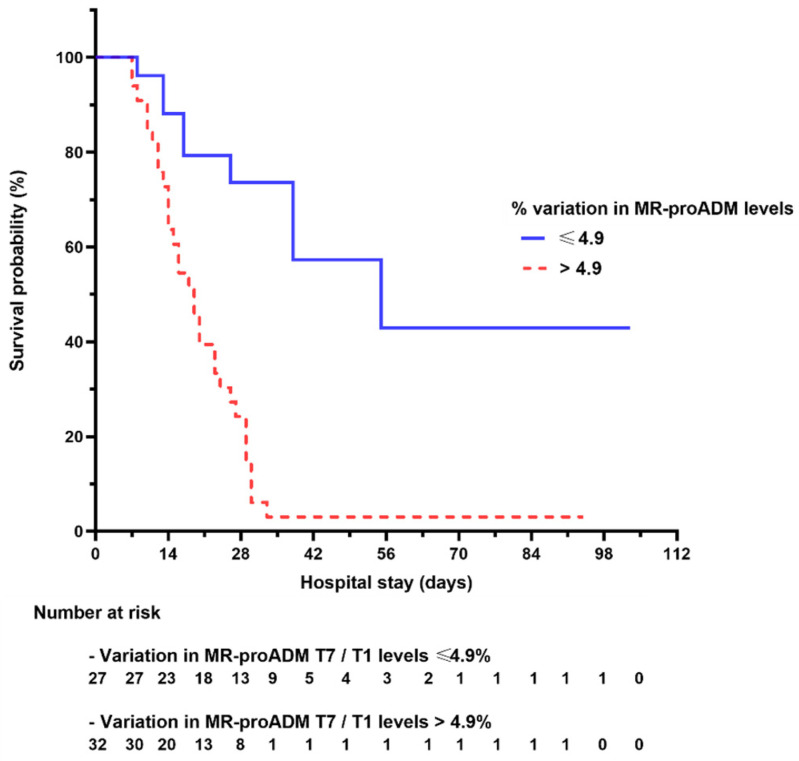
In-hospital mortality in patients with a percentage variation of mid-regional pro-adrenomedullin (MR-proADM) between T1 (first 48 h after the moment of admission to the ICU) and T7 (between 6 and 8 days after the moment of admission to the ICU) > 4.9% or ≤ 4.9%. ICU: intensive care unit.

In particular, an increase in the T7/T1 ratio > 4.9% carried a more than eight-fold risk of ICU mortality (HR 8.633, 95% CI 3.549–21.002, *p* < 0.001) and four-fold chance of in-hospital mortality. Of note, this result was confirmed in all the regression models evaluated considering demographic features, metabolic comorbidities, inflammatory indices at T1 or immunomodulation therapies (HR 4.417, 95% CI 2.079–9.385, *p* < 0.001) (Table 2, Figure 4). Most of all, this result maintained significance even taking into consideration all the traditional mortality scores (HR 4.958, 95% CI 2.217–11.086, *p* < 0.001).

### 3.2. Superinfection

Three patients (4.4%) presented bacterial coinfection before entering the ICU. Seventeen patients (26.2%) underwent superinfection during the first week in the ICU, while 13 (19.1%) patients were superinfected later (median time to superinfection of 6 [3–10] days, range 9–39 days). Both PCT and CRP evaluated at T1 were not able to predict superinfection within the first week after ICU admission even taking into account concomitant corticosteroid treatment. Conversely, MR-proADM evaluated at T1 resulted in being significantly associated with bacterial superinfection within one week, even considering the same ongoing treatment (Table 2). Finally, such an event was predicted by the SOFA score calculated upon ICU access (HR 1.173, 95% CI 1.019–1.350, *p* = 0.026). Both MR-proADM at T1 and the SOFA score did not maintain statistical significance when adjusted for the need for vv-ECMO.

Among superinfected patients, 16 (53.3%) developed septic shock. Patients who developed septic shock showed increasing copeptin values during their ICU stay, with a significant difference in the T7/T1 ratio compared to patients who did not develop this complication (*p* = 0.041); no biochemical parameter analyzed at T1, T3 or T7 proved to be useful in predicting the occurrence of septic shock during the ICU stay.

### 3.3. Renal Replacement Therapy

Eleven patients (16.2%) underwent RRT during hospitalization, and the median time to RRT was 8 [2.75–17] days. Patients requiring RRT presented eGFR levels significantly lower than controls at every time point (T1: 68.0 [15.73–90.58] vs. 96.12 [82.93–110.15] mL/min/1.73 m^2^, *p* = 0.001; T3: 47.62 [19.85–88.78] vs. 98.50 [88.25–113.40] mL/min/1.73 m^2^, *p* < 0.001; T7: 23.10 [15.80–86.90] vs. 97.93 [86.90–114.45] mL/min/1.73 m^2^, *p* < 0.001).

The need for RRT was significantly predicted by the SAPS II score (HR 1.055, 95% CI 1.008–1.105, *p* = 0.022) as well as by both copeptin and MR-proADM levels measured at T1 (HR 1.007, 95% CI 1.002–1.012, *p* = 0.006; HR 1.238, 95% CI 1.122–1.367, *p* < 0.001, respectively). The significance for every predictor, however, was lost when adjusted for admission eGFR values. When taking into account any degree of kidney impairment at the time of hospitalization (i.e., eGFR < 90 mL/min/1.73 m^2^), copeptin levels at T1 >23.4 pmol/L remained significant predictors of the subsequent need for RRT, even if the analysis was adjusted for SAPS II score (HR 5.305, 95% CI 1.047–26.874, *p* = 0.044) (Table 2).

### 3.4. Veno-Venous Extracorporeal Membrane Oxygenation

A total of 8 patients (11.8%) were already on vv-ECMO at the time of ICU entry, while 16 patients (23.5%) underwent vv-ECMO in ICU during the first week [range 0–5 days]; in particular, 12 out of 16 (75%) patients underwent vv-ECMO right upon ICU admission.

No differences were observed regarding the endocrine biomarkers at any time point between patients undergoing vv-ECMO support and those not. Furthermore, only 2 patients out of 24 could be weaned from vv-ECMO due to clinical improvement; therefore, in consideration of the small number of patients, it was not possible to look for correlations with any measured analyte.

### 3.5. Invasive Mechanical Ventilation

Thirty-four patients (50%) had already undergone IMV at the time of admission to the ICU, while the vast majority of those who had not (30 out of 34, 88.2%) were intubated in the first days of hospitalization (median time without IMV 1 day). The median duration of IMV in the entire population was 13 [7–25] days. Given the small number of non-ventilated patients, it was not possible to look for correlations between the variables analyzed and the need for IMV. Twenty-four patients, after initial clinical improvement, were weaned from IMV (median time to weaning 8 [5–13] days). The weaning from IMV due to clinical improvement was independently predicted by the MR-proADM T7/T1 ratio, even if adjusted for demographic features, metabolic comorbidities, inflammatory indices evaluated at T1, immunomodulation therapies and MuLBSTA score (Table 2). No significant correlation was observed with any other biomarker analyzed.

### 3.6. Pronation

A total of 49 patients (72%) required pronation as rescue treatment during their ICU stay. The need for pronation was significantly predicted by the MuLBSTA classification determined at ICU entry even if adjusted for demographic features and metabolic comorbidities (OR 1.225, 95% CI 1.015–1.478, *p* = 0.034). In particular, subjects with MuLBSTA score ≥ 12 upon ICU access were more likely to be pronated during their hospital stay (OR 4.822, 95% CI 1.489 to 15.611, AUC 0.685, 95% CI 0.561–0.792, *p* = 0.009). No other correlation was identified between the other scores calculated upon ICU admission and the need for pronation.

## 4. Discussion

The results obtained in the present study, a predefined secondary analysis of a larger cohort previously described [14,25], demonstrate and confirm the role of MR-proADM [14,25,32] in predicting adverse outcomes in patients requiring intensive care assistance due to COVID-19 infection. Moreover, our data suggest the potential value of assessing copeptin and MR-proANP, either independently or in combination with commonly used mortality scores, in stratifying and predicting adverse outcomes in patients requiring intensive care assistance. In particular, it is possible to predict ICU and in-hospital mortality as well as the need for RRT.

Of note, the population analyzed was largely composed of male subjects with a high prevalence of metabolic comorbidities (i.e., diabetes mellitus, arterial hypertension and obesity). This finding confirms the greater propensity of this sex to develop severe or critical forms of COVID-19 [33] as well as the role of metabolic syndrome as a well-known and relevant risk factor for a more serious course of such disease [34]. With regard to mortality, our data are slightly higher than others available in the literature (69.1 vs. 41.6% [34.0–49.7] [35]) with a median survival time of 16 days. This fact could be partly explained by the particular severity of the patients admitted to our reference center during the first two waves of COVID-19 infection.

In our cohort, MR-proADM levels were significantly different at any time point between survivors and non-survivors with an increasing trend observed in the latter and a decreasing one in the former. This variation, unlike single marker determinations, could predict both ICU and in-hospital mortality in patients hospitalized for COVID-19 even for minimal increases (>4.9%) after admission to the ICU. These results were also adjusted for the most common metabolic comorbidities, as well as for immunomodulation therapies and inflammatory parameters commonly used in clinical practice, such as CRP and PCT. Indeed, MR-proADM has shown to be a more accurate biomarker compared to PCT in septic patients for disease severity and mortality risk stratification [25,36]. Similarly, the reduction in MR-proADM values compared to baseline was significantly associated with the probability of weaning from IMV, probably reflecting how the decline of this marker over time was associated with a better overall clinical outcome. In this regard, it is likely that the effectiveness of MR-proADM in predicting mortality is due to the important role of ADM in modulating the systemic inflammatory response, which is often altered in patients hospitalized for SARS-CoV-2 infection [37]. As a matter of fact, several studies in the literature [20,21,24,38,39,40,41,42] have described the high prognostic power of MR-proADM in patients with community-acquired pneumonia or sepsis or PE, which are frequent complications, as well as possible causes of death, in patients with SARS-CoV-2 infection. Likewise, MR-proADM itself has been extensively shown to be associated with increased mortality in patients hospitalized for COVID-19 regardless of other known cardiovascular risk factors [7,14,25,32,43].

As expected, our study confirmed that in-hospital mortality can be accurately predicted using the SAPS II classification or the SOFA score as well. In our analysis, the increase in the MR-proADM T7/T1 ratio was associated with a higher risk of in-hospital mortality, even with all of the prognostic scores being taken into consideration [20]. In this regard, it is likely that MR-proADM closely reflects the extent of endothelial vascular damage caused by both SARS-CoV-2 infection and multiorgan failure, thus effectively introducing a variable that is not otherwise assessed in such scores. Certainly, endothelial damage is a typical consequence of a cytokine storm, which in turn can be considered a cross-sectional epiphenomenon related not only to severe COVID-19 infection, but also to most previous and probably future pandemics in history [44].

Finally, though reported by only one study to date [11], our analysis also confirms that ICU mortality may be predicted by MR-proANP measured upon hospital admission, even though significance was lost if adjusted for PCT and corticosteroid treatment. In the study by Kaufmann et al. [11], however, PCT values were not measured, and therefore, the prognostic role of MR-proANP in the mortality of patients with sepsis secondary to severe COVID-19 infection needs to be further investigated.

Bacterial superinfections represent one of the most frequent complications of COVID-19 [45]. They affected nearly half of the patients in the ICU and generally developed within the first 10 days of hospitalization. Superinfected patients require isolation and the use of more personal protective equipment than usual. This considered, recognizing patients at increased risk could allow preventive actions to counter the spread of pathogens more effectively. Although the SOFA score and MR-proADM levels were found to predict superinfection occurrence during the first week in the ICU, these correlations were lost when adjusted in special populations, such as patients requiring vv-ECMO support. ECMO itself might represent an important risk factor for the development of superinfection, as it involves the use of two venous accesses throughout the duration of treatment [46], as it usually normally causes immunological and endothelial alterations related to the causative disease, but also to the support itself. In the current cohort, however, no significant differences were observed in the median values of the three biomarkers analyzed between patients who required vv-ECMO and those who did not. The small size of our sample may have influenced these data, which appear particularly interesting to be further investigated in the future. In fact, if confirmed, they could suggest a smaller interference than expected in endothelial biomarker production and release despite such an invasive therapeutic procedure.

One more area in which the aforementioned biomarkers were effective was in predicting the need for RRT. In particular, both MR-proADM and copeptin were significant predictors of the subsequent need for RRT, though significance was lost when adjusted for eGFR values on admission. Indeed, both biomarkers have been shown to be elevated in the early stages of CKD and to be correlated with renal disease progression in both diabetic and non-diabetic patients [47,48,49,50,51]. Moreover, MR-proADM itself has already been suggested as a possible marker for properly identifying COVID-19 patients at increased risk of undergoing RRT during hospital stays [52]. Elevated values of these biomarkers at the time of ICU entry thus probably reflect some degree of early renal impairment, since the clearance of both molecules, especially copeptin [53], involves the kidney. Nevertheless, our data indicate that an early copeptin level > 23.4 pmol/L correlates with a more than five-fold higher risk of requiring RRT during hospitalization, when considering patients with any reduction in eGFR, even if minimal, thus improving the predictive role of SAPS II.

Finally, regarding the need for pronation, we documented a significant correlation with the MuLBSTA class recorded on admission to the ICU. This score, created to predict mortality in patients with viral pneumonia, is composed of “low-risk” and “high-risk” classes, and subjects who fell into the latter were more likely to need pronation. Indeed, it was already shown that in patients with COVID-19, MuLBSTA classification significantly improved performance in predicting disease behavior, so our results confirm the data obtained by Iijima et al. [54].

All this considered, the primary strength of our study lies in the simultaneous consideration of multiple traditional severity scores and the serial evaluation of three promising endocrine biomarkers. This integration extends our previous findings [14,25], which were limited to the analysis of the prognostic value of MR-proADM. Additionally, we established an excellent cut-off for this analyte in predicting ICU and in-hospital mortality, based on the prospective assessment of its trend in the ICU. Finally, compared to our previous data [14,25], each predictor underwent a more robust evaluation through Cox multivariate regression analyses.

On the other hand, our study presents some limitations. Firstly, it is a monocentric experience evaluating a rather small cohort of patients. In addition, it refers to a highly complex university center, receiving critical patients as secondary hospitalization for vv-ECMO support. Finally, the turnover of patients during a pandemic period may have resulted in a selection bias, thereby leading to a lack of a comparison population.

## 5. Conclusions

MR-proADM, copeptin and MR-proANP significantly correlate with the main adverse outcomes in severe COVID-19 patients admitted to the ICU during the first waves, likely deserving to be evaluated in the future together with the SOFA, SAPS II and MuLBSTA prognostic scores. Considering the role of both cytokine storm and endothelial damage in various severe infectious diseases, not limited to those induced by SARS-CoV-2, these findings could potentially be applicable to other severe viral infections. Although the early assessment and time course of these biomarkers might be helpful in better defining the prognosis and risk of complications, further studies are needed to accurately integrate them into these prognostic risk scores, assigning an appropriate weight to each biomarker.

## Figures and Tables

**Table 1:** Demographic and clinical characteristics of the enrolled patients at baseline. Data are expressed as median and interquartile range (IQR) or *n* (%).

Patients’ Characteristics	Overall (*n* = 68)	Survivors (*n* = 21)	Non-Survivors (*n* = 47)	*p*-Value
Age, years, *median (IQR)*	63 (56–71)	68 (54.5–71)	63 (53–73.75)	0.577
Gender, male, *n* (%)	52 (76.5)	18 (85.7)	34 (72.3)	0.233
BMI, kg/m^2^, *median (IQR)*	28.2 (26.6–33.9)	27.8 (27.0–34.2)	28.4 (26.4–33.9)	0.931
Patients transferred from other ICUs, *n* (%)	33 (48.5)	8 (38.1)	25 (53.2)	0.347
Diabetes mellitus, *n* (%)	12 (17.6)	4 (19.05)	8 (17.02)	0.841
Arterial hypertension, *n* (%)	45 (66.2)	14 (66.7)	31 (65.6)	0.955
Obesity, *n* (%)	43 (63.2)	14 (66.7)	29 (61.7)	0.697
SOFA score, *median (IQR)*	8 (5–10.5)	7 (4.75–8)	8 (5–11)	**0.028**
MuLBSTA score, *median (IQR)*	13 (10.5–13.0)	12 (9.25–13.0)	13 (11–15)	0.079
SAPS II score, *median (IQR)*	52 (41–60)	44 (31.5–59.5)	54 (43–60)	0.056
IMV, *n* (%)	64 (94.1)	19 (90.5)	45 (95.7)	0.397
vv-ECMO, *n* (%)	24 (35.3)	2 (9.5)	22 (46.8)	**0.003**
Pronation, *n* (%)	49 (72.1)	13 (61.9)	36 (76.6)	0.216
Superinfection, *n* (%)	30 (44.1)	3 (14.3)	27 (57.4)	**<0.001**
Superinfection within the first week in ICU, *n* (%)	17 (26.2)	2 (9.5)	15 (34.1)	**0.037**
Septic shock (amongst patients with previous superinfection), *n* (%)	16 (53.3)	1 (6.25)	15 (93.75)	0.472
Renal replacement therapy, *n* (%)	11 (16.2)	1 (4.8)	10 (21.3)	0.090
ICU mortality, *n* (%)	42 (61.8)			
In-hospital mortality, *n* (%)	47 (69.1)			
ICU LOS, days, *median (IQR)*	13 (7–22)	10 (5.75–18.25)	14 (8.5–24.75)	0.151
Hospital LOS, days, *median (IQR)*	20 (13–30)	30 (20.25–42.5)	17 (11.25–26.75)	**0.001**
Corticosteroid treatment, *n* (%)	51 (75)	15 (71.4)	36 (76.6)	0.652
Tocilizumab, *n* (%)	10 (14.7)	1 (4.8)	9 (19.1)	0.125
Hydroxychloroquine, *n* (%)	15 (22.1)	5 (23.8)	10 (21.3)	0.871

The numbers in bold indicate significant values (*p* < 0.05). Abbreviations: BMI: body mass index; ICU: intensive care unit; LOS: length of stay; IMV: invasive mechanical ventilation; vv-ECMO: veno-venous extracorporeal membrane oxygenation.

**Table 2:** Multivariate regression models used to predict the different outcomes of interest.

Outcome	Predictor	Model Adjustment	HR	95% CI	*p*-Value
ICU mortality	MR-proANP at T1	DM, hypertension, obesity	1.005	1.001–1.011	**0.042**
		Sex, age	1.005	1.001–1.010	**0.049**
		CRP at T1, corticosteroid	1.006	1.001–1.012	**0.025**
		PCT at T1, corticosteroid	1.006	0.999–1.011	0.056
		Tocilizumab, corticosteroid, hydroxychloroquine	1.006	1.001–1.011	**0.035**
In-hospital mortality	MR-proADM (T7 − T1)/T1 > 4.9%	DM, hypertension, obesity	5.223	2.434–11.209	**<0.001**
		Sex, age	5.074	2.376–10.838	**<0.001**
		CRP, PCT at T1	5.230	2.414–11.331	**<0.001**
		Tocilizumab, corticosteroid, hydroxychloroquine	4.417	2.079–9.385	**<0.001**
		SOFA, SAPS II, MuLBSTA	4.958	2.217–11.086	**<0.001**
Superinfection within 1 week in ICU	MR-proADM at T1	Corticosteroid	1.113	1.014–1.220	**0.024**
RRT	Copeptin > 23.4 pmol/L	SAPS II, eGFR sec CKD-EPI < 90 mL/min/1.73 m^2^	5.305	1.047–26.874	**0.044**
IMV weaning	MR-proADM T7/T1 ratio	DM, hypertension, obesity	0.988	0.979–0.997	**0.012**
		Sex, age	0.989	0.980–0.998	**0.015**
		CRP, PCT at T1	0.983	0.972–0.994	**0.002**
		Tocilizumab, corticosteroid, hydroxychloroquine	0.987	0.977–0.998	**0.016**
		MuLBSTA	0.988	0.979–0.998	**0.019**

The numbers in bold indicate significant values (*p* < 0.05). Abbreviations: HR: hazard ratio; CI: confidence interval; ICU: intensive care unit; MR-proANP: mid-regional pro-atrial natriuretic peptide; MR-proADM: mid-regional pro-adrenomedullin; T1: first 48 h after the moment of admission to the ICU; DM: diabetes mellitus; CRP: c-reactive protein; PCT: procalcitonin; T7: between 6 and 8 days after the moment of admission to the ICU; RRT: renal replacement therapy; eGFR: estimated glomerular filtration rate; IMV: invasive mechanical ventilation.

**Table 3:** Laboratory parameters at the different time points in survivors and non-survivors. Data are presented as median and interquartile range (IQR).

Laboratory Parameters	T1 (68 Patients)		*p*-Value	T3 (68 Patients)		*p*-Value	T7 (59 Patients)		*p*-Value
	Survivors *(n* = 21)	Non-Survivors (*n* = 47)		Survivors (*n* = 21)	Non-Survivors (*n* = 47)		Survivors (*n* = 18)	Non-Survivors (*n* = 41)	
MR-proADM (nmol/L), *median (IQR)*	0.93 (0.75–1.31)	1.31 (0.86–2.12)	**0.019**	0.96 (0.76–1.45)	1.43 (0.96–2.12)	**0.040**	0.84 (0.63–1.07)	2.12 (1.27–2.86)	**<0.001**
MR-proANP (pmol/L), *median (IQR)*	56.48 (21.35–95.71)	61.80 (37.90–101.58)	0.219	55.75 (21.60–105.43)	86.63 (49.01–119.95)	0.195	67.03 (21.60–112.0)	59.68 (32.14–114.60)	0.559
Copeptin (pmol/L), *median (IQR)*	13.90 (8.27–38.78)	17.13 (6.0–29.65)	0.638	13.70 (5.76–20.03)	17.70 (6.27–33.85)	0.333	19.84 (5.64–40.10)	24.50 (13.0–54.70)	0.189
CRP (mg/L), *median (IQR)*	117.90 (47.33–173.58)	117.6 (52.45–220.63)	0.582	65.10 (30.23–108.75)	83.0 (33.73–155.23)	0.609	61.95 (18.40–119.20)	110.0 (24.78–171.55)	0.069
PCT (µg/L), *median (IQR)*	0.12 (0.09–0.74)	0.39 (0.15–1.53)	0.051	0.15 (0.06–1.41)	0.34 (0.14–1.26)	0.104	0.23 (0.10–0.97)	0.50 (0.16–1.04)	0.189
eGFR (mL/min/1.73m^2^), *median (IQR)*	96.10 (88.40–103.80)	89.0 (72.70–113.60)	0.791	97.5 (90.35–103.90)	90.9 (72.05–114.60)	0.894	96.25 (86.90–111.20)	92.70 (48.0–114.45)	0.736

The numbers in bold indicate significant values (*p* < 0.05). Abbreviations: T1: first 48 h after the moment of admission to the ICU; T3: between 2 and 4 days after the moment of admission to the ICU; T7: between 6 and 8 days after the moment of admission to the ICU; MR-proADM: mid-regional pro-adrenomedullin; MR-proANP: mid-regional pro-atrial natriuretic peptide; CRP: c-reactive protein; PCT: procalcitonin; eGFR: estimated glomerular filtration rate.

## Data Availability

The datasets generated during and/or analyzed during the current study are not publicly available but are available from the corresponding author on reasonable request.
